# Deep learning‐based CNN method for fiducial marker detection in kilovoltage X‐ray images for liver tumor motion monitoring

**DOI:** 10.1002/mp.70691

**Published:** 2026-10-04

**Authors:** Frances Kan, Freeman Jin, Adam Mylonas, Benjamin Zwan, Trang Nguyen, Trevor Moodie, Nicholas Hardcastle, Sau Fan Liu, Daniel Mason, Tim Wang, Yoo Young Lee, Paul Keall, Chandrima Sengupta

**Affiliations:** ^1^ Faculty of Medicine and Health, Image X Institute The University of Sydney Sydney New South Wales Australia; ^2^ Department of Radiation Oncology Central Coast Cancer Centre Gosford New South Wales Australia; ^3^ Department of Radiation Oncology Crown Princess Mary Cancer Centre Westmead New South Wales Australia; ^4^ Department of Radiation Oncology Peter MacCallum Cancer Centre Melbourne Victoria Australia; ^5^ Department of Radiation Oncology Princess Alexandra Hospital Woolloongabba Queensland Australia; ^6^ Department of Radiation Oncology Nepean Cancer and Wellness Centre, Nepean Hospital Kingswood New South Wales Australia

**Keywords:** deep learning, real‐time IGRT for liver SABR, tumor motion

## Abstract

**Background:**

During radiation therapy, liver tumor motion can reduce dose delivery accuracy and increase irradiation of adjacent healthy tissues. Because liver tumors are difficult to visualize directly on kilovoltage (kV) X‐ray images, fiducial markers are commonly implanted as surrogates for tumor position during treatment. Conventional marker‐segmentation approaches such as template matching can lose accuracy when markers are obscured by bone, surgical clips, or stents. Although Convolutional Neural Network (CNN)‐based approaches have shown strong performance for image analysis, their application has been constrained to more static organs such as the prostate or has used single‐center and vendor‐specific datasets, limiting assessment of model transportability across different institutions and anatomic sites.

**Purpose:**

The aim of this study was to develop, integrate, and evaluate a CNN‐based method for fiducial marker detection in kV X‐ray images to support real‐time image‐guided radiation therapy (IGRT) during liver radiotherapy using a multi‐institutional, multi‐platform dataset spanning both large and fast free‐breathing motion and slow and small breath‐hold motion.

**Methods:**

A compact Convolutional Neural Network (CNN) was trained on 314,625 kilovoltage (kV) X‐ray images encompassing 28 patients from the multi‐institutional TROG 17.03 LARK clinical trial (NCT02984566). The model was validated using a hold‐out set of 31,463 images (10%) from the same cohort of 12 patients and 55 treatment fractions, spanning three centers and three respiratory motion‐management techniques. The CNN was tested on 4184 images from 16 patients and 55 fractions across three centers and three motion‐management techniques. The ground truth for testing was manually segmented marker positions from every 10 degrees of gantry rotation for each fraction of each test patient. Based on AAPM guidelines (TG147 and TGB135.B), feasibility for clinical implementation was predefined as > 95% of marker positions being within 2 mm of the ground truth position in each dimension, and processing time less than 150 ms per image using a simulated real‐time Kilovoltage Intrafraction Monitoring (KIM) framework. Additional evaluation metrics included sensitivity, specificity, and the area under the precision‐recall curve (AUC).

**Results:**

For the unseen test patient data, the marker position was segmented by the CNN within 2 mm in 95.4% of frames on the X axis and 97.6% of frames on the Y axis, meeting AAPM criteria. Sensitivity reached 97.76%, specificity was 99.94%, and the AUC was 0.9964. No statistically significant difference in localization error was observed between breath‐hold and free‐breathing treatments, although this comparison was limited by the small number of free‐breathing patients. Localization error differed significantly between Varian and Elekta linacs due to imbalance within the dataset. The processing time for each image was 50–60 ms using a NVIDIA GeForce RTX 3070 GPU.

**Conclusion:**

A CNN‐based method for fiducial marker detection in the liver was developed and evaluated on multi‐institutional kV X‐ray images spanning free‐breathing and breath‐hold motion, as well as Varian and Elekta linac platforms. The method satisfied the predefined feasibility criteria for positional accuracy and computation time, supporting future translation toward clinical implementation.

## 1 INTRODUCTION

In the high precision treatment of metastatic or primary liver cancers, stereotactic ablative radiation therapy (SABR) has been applied successfully to deliver high doses of radiation to tumors not eligible for surgical intervention.^1^ However, the intrinsic challenge of SABR is accounting for the motion of the liver induced by patient respiration and movement of upper abdominal organs, which can substantially reduce the radiation dosage to tumor sites and increase risk of radiation‐induced liver toxicity in healthy tissues.^2^, ^3^ In a single respiratory cycle, the liver is known to move as much as 50 mm in the cranio‐caudal direction^3^ and depending on the location of the target lesion, a prescription dose reduction is required to reduce excess dose to healthy radiosensitive organs such as bowels, esophagus, stomach and liver itself.^4^, ^5^ Real‐time image‐guided radiation therapy (IGRT) presents a potential solution by monitoring intrafraction motion to increase precision in delivering high radiation doses to target tissue.^2^, ^4^, ^6^, ^7^, ^8^, ^9^

Commercial systems have implemented highly specialized solutions such as the CyberKnife,^10^, ^11^ Radixact^12^ and gimbaled Vero,^13^ which use integrated and continuous imaging systems tracking implanted fiducials to enable precise targeting of treatment. Additionally, MRI‐guided radiation therapy systems allow for direct visualization of the tumor such that treatment can be gated following liver tumor movement.^14^ However, to reduce overhead and increase accessibility of these approaches, conventional linear accelerators (linacs) have been used for real‐time motion management using kilovoltage (kV) imaging.^4^, ^9^

On standard linacs, intra‐fraction 2D image guidance is available using the commercially available Triggered Imaging system at a limited imaging frequency.^15^, ^16^ However, the 2D imaging lacks the motion component along the X‐ray beam axis and therefore limits the ability to perform 6D monitoring. To address this limitation, a real‐time 6D internal target monitoring technology named Kilovoltage Intrafraction Monitoring (KIM) was developed and clinically implemented for prostate^17^ and liver.^4^

Real‐time motion monitoring^4^, ^8^, ^18^, ^19^, ^20^ in kV images typically requires surgically implanted markers as surrogates of the tumor motion due to poor soft tissue contrasts. Existing approaches to marker detection leverage traditional approaches for image detection such as template matching,^4^ which relies on the consistency in appearance of markers to locate target tissue in new images. However, the development of a template requires knowledge of marker properties obtained through the creation of a predefined template of marker appearance which is generated during a learning period that subjects patients to an additional imaging dose. Further, the accuracy of template matching is reduced when markers are obscured by high density materials, such as bone, surgical clips, and stents.^21^

CNN‐based methods have shown strong performance for image analysis in IGRT and may overcome several limitations of template matching.^8^, ^22^, ^23^, ^24^ However, the liver's association with multiple dynamic structures results in large and complex motion patterns that complicate detection^1^, ^3^, ^25^ when compared to more static organs such as the prostate which experience a magnitude of motion that is 2–3 times smaller.^3^, ^23^ In addition, marker visibility in liver imaging can be degraded by bone, surgical clips, and stents, and robust CNN training requires large, annotated datasets spanning these variations for training, validating, and testing.^6^, ^26^ Previous applications of marker detection as a surrogate for tissue motion have been confined to slow and small prostate motion^8^, ^23^ or single center datasets,^19^, ^22^ and are characterized by the absence of a large, annotated image dataset leveraging cross‐clinic cooperation that is necessary to increase transportability of models across different institutions. Moreover, all previous datasets were limited to a single vendor platform, either Varian^8^, ^22^ or Elekta.^23^ Therefore, multi‐institutional and multi‐platform data incorporating multiple breathing modes provide an opportunity to investigate CNN performance under clinically heterogeneous liver SABR imaging conditions.

This study extends a previously developed compact deep‐learning marker‐detection approach from prostate tracking^8^ to the more challenging liver SABR setting using clinically acquired kV X‐ray images from the multi‐institutional TROG 17.03 Liver Ablative Radiotherapy using Kilovoltage intrafraction monitoring (LARK) clinical trial (NCT02984566) (Figure 1). The novelty of this work lies in the integration of a lightweight CNN‐based candidate tile classifier into the real‐time KIM framework,^4^ and its evaluation using a multi‐center liver dataset encompassing two linear accelerator platforms from different vendors (Varian and Elekta), multiple motion management techniques including free‐breathing and breath‐hold treatments, and manually verified marker positions. The objective was to assess whether this CNN‐KIM integration could maintain clinically feasible marker‐detection accuracy and latency without additional template‐learning imaging or prior specification of marker appearance.^4^

**FIGURE 1:**
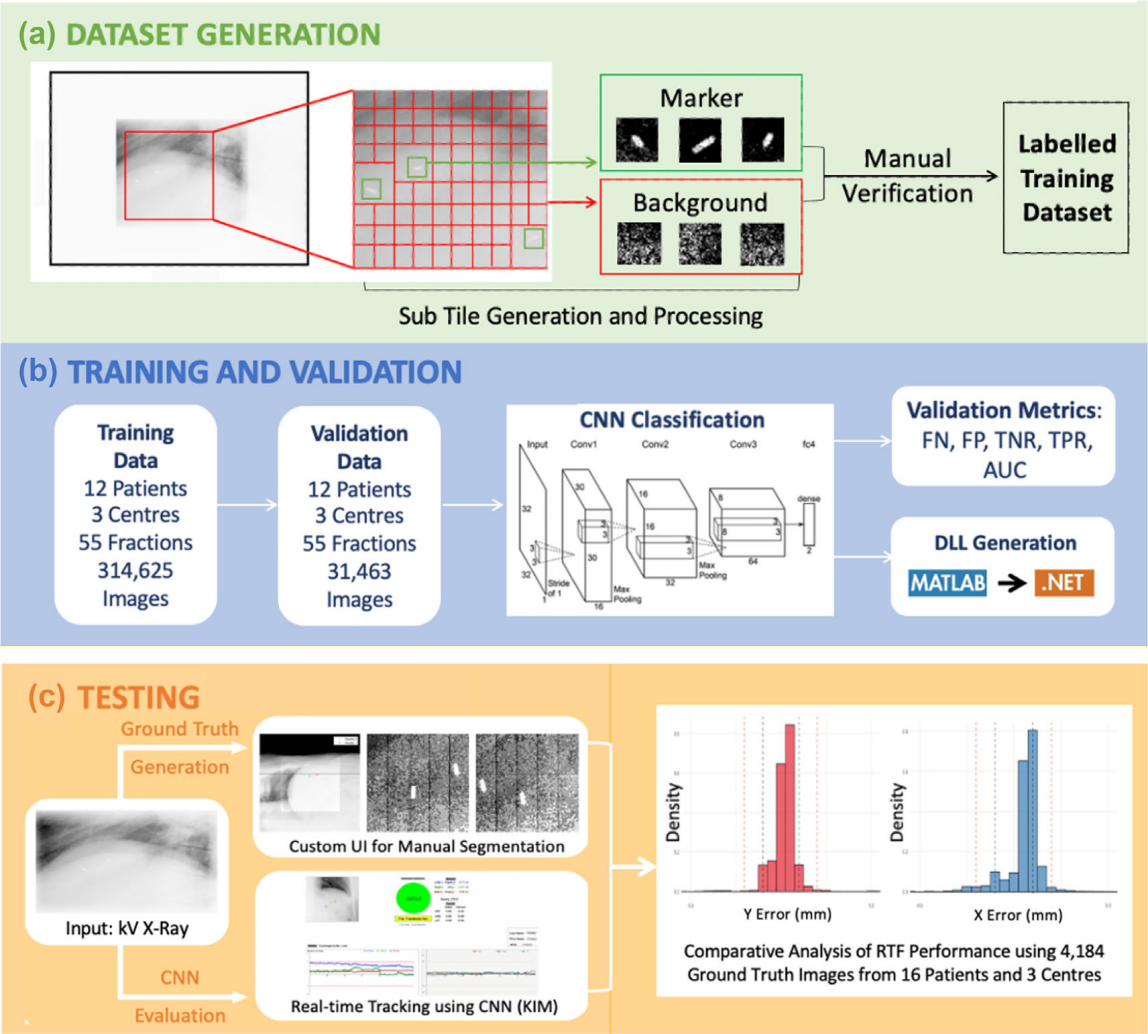
Schematic overview of the workflow used for CNN training and validation on kV X‐ray images from 12 patients and testing on images from 16 patients.

## 2 MATERIALS AND METHODS

The study schema for training, validation, and testing of the CNN‐based method is shown in Figure 1.

### 2.1 Data acquisition

Images were sourced from the multi‐institutional, multi‐platform TROG 17.03 LARK clinical trial (NCT02984566), which evaluated real‐time image‐guided liver SABR using implanted cylindrical gold markers as surrogates for tumor motion using template matching based detection with the KIM system.^4^ Training and validation data comprised intrafraction kV X‐ray images from 55 fractions across 12 patients treated at three centers. In total, 314,625 X‐ray images were used for CNN training and 31,463 images were used for validation. Testing data comprised 4184 manually annotated images from 16 unseen patients and 55 fractions across three treatment centers, including breath‐hold and free‐breathing acquisitions. The motion‐management strategies included deep‐inspiration breath‐hold (*n* = 3), end‐expiration breath‐hold (*n* = 9), and free breathing (*n* = 4). Patients received one of four fractionation schedules: 3 × 17 Gy, 5 × 10 Gy, 5 × 8 Gy, or 5 × 5.5 Gy. The study cohort had a median age of 67 years (range 32–78), diagnoses included hepatocellular carcinoma and liver metastases, and ECOG performance scores ranged from 0–1. Twelve patients were treated using Varian TrueBeam linear accelerators and four were treated using Elekta Versa HD linear accelerator.

### 2.2 Convolutional neural network architecture

Convolutional Neural Networks (CNNs) were selected for their ability to extract spatial and hierarchical features, enabling the identification of markers within kV images.^7^ As the performance of transfer learning leveraging pretrained CNNs with increased convolution layers was previously determined to be comparable to the compact CNN model,^8^ the chosen architecture retained three convolutional layers, each followed by batch normalization and ReLU activation. This lightweight design ensured inference speeds were compatible with real‐time intrafraction monitoring, and reduced parameter space enhanced model robustness by minimizing the risk of overfitting to center‐specific noise. Max pooling after the first two convolutional layers reduced spatial dimensionality and supported feature extraction. A fully connected layer followed by a softmax output layer performed binary classification of marker versus background tiles.

### 2.3 Data pre‐processing for training

The patient images were previously subclassified to fraction levels and were pre‐processed in MATLAB r2024a by cropping to a standard, centered region of 120 x 120 mm to isolate the anatomical region of interest around the tumor, enhancing training efficiency.

Existing marker positions generated from a template‐matching algorithm developed within the KIM framework^4^ were initially used to determine the pixel positions of markers present in the kV images to define positive sub images in the training dataset for the CNN. To reduce the potential for errors in the ground truth, only marker locations generated for X‐ray images taken from patients where the KIM algorithm performed detection accurately were retained. Additionally, in positive sub images, the presence of centered markers was manually verified prior to addition within the training dataset. This allowed for the representation of challenging detections in heterogeneous regions of marker occlusion or variable background intensities that interfere with traditional marker recognition techniques.

Subregions of 32 × 32 pixels centered on the marker centroid were cropped as positive tiles, and the remaining image area was subdivided into negative background tiles. The tiles were normalized using mean and standard deviation normalization followed by min‐max scaling to the [0, 1] range.

To remove partial markers, any background tiles generated via the sliding window approach that overlapped with any section of previously generated marker tiles were discarded to avoid partial inclusion of markers. Images containing multiple markers were included in the positive subset, with one specific marker being identified as the center. Artifacts such as surgical clips, delineations of Real‐time Position Management (RPM) blocks, and vertebral elements which had previously been incorrectly identified as markers by the template matching algorithm^4^ were retained in background images to assist in the reduction of false positives.

Eight rotations and inversions were applied to both positive and negative tiles to increase representation of different marker orientations. All positive subtiles were subsequently visually verified.

### 2.4 CNN training protocol

The network was trained using stochastic gradient descent with momentum on an NVIDIA RTX A6000 GPU. Mini‐batches of 128 were used. Learning rates of 0.0001 and 0.001 were compared with a momentum of 0.9. Because the higher learning rate yielded similar training duration but lower validation accuracy, models trained with a learning rate of 0.0001 were retained for implementation. Early stopping terminated training if validation accuracy did not improve for three consecutive epochs to reduce overfitting and improve computational efficiency. The final model trained for 17 epochs.

Tile‐level classification performance was first evaluated in MATLAB using unseen images from five patients at one center with relatively high image noise and common artefacts, including surgical clips and RPM blocks. The trained CNN classified each subtile as marker (positive) or background (negative), and predictions were compared with the reference labels used for data generation. Sensitivity, specificity, and area under the receiver operating characteristic curve (AUC) were used to evaluate performance. For visual review, predictions were sorted into true‐positive, false‐positive, true‐negative, and false‐negative categories.

### 2.5 Evaluation using real‐time radiation therapy framework (KIM)

#### 2.5.1 CNN integration within KIM

To enable real‐time marker detection for clinical deployment, the trained CNN was integrated into the KIM platform for marker detection during liver SABR treatments using patient images excluded from the training set. The integration involved creating a dynamic‐link library (DLL) using MathWorks Builder to package the MATLAB‐trained CNN into a .NET assembly compatible with the Visual Studio 2022 C# environment. Analogues to functions for normalization and dynamic contrast adjustment within the MATLAB dataset generation script were created within the C# framework to standardize image inputs between the two interfaces. Real‐time detection was run using a NVIDIA GeForce RTX 3070 GPU. The CNN was implemented as a binary candidate‐tile classifier within the KIM marker‐detection workflow (Figure 2). Specifically, candidate sub‐images generated within the expected marker search region were classified as either marker‐containing or background. Positive candidate detections were then converted into marker centroid positions and passed to the existing KIM tracking framework to generate marker trajectories. Here, the role of the CNN was to replace the template‐matching marker‐detection step in KIM, while the downstream KIM tracking framework was retained.

**FIGURE 2:**
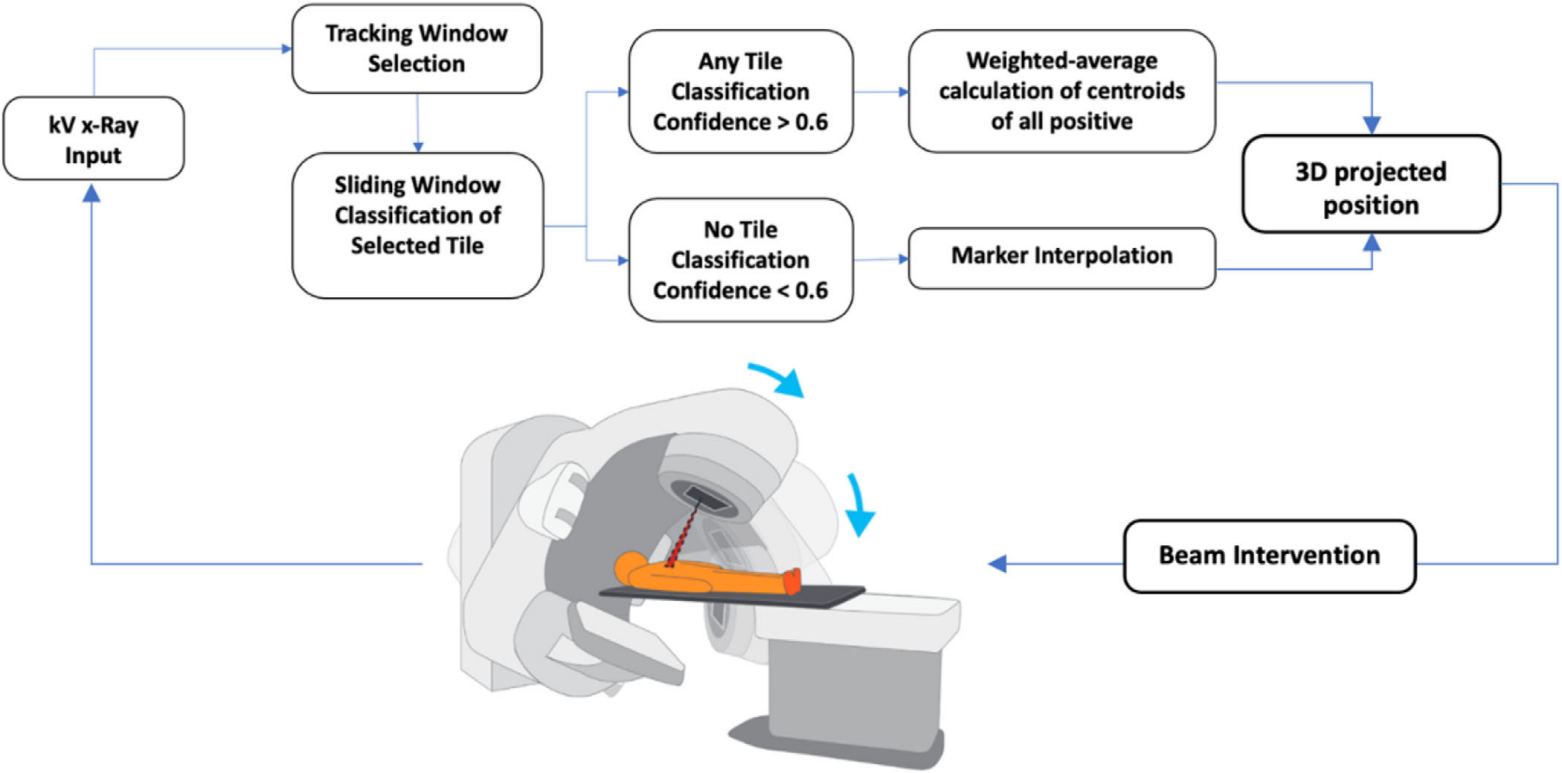
CNN‐assisted marker detection workflow within the KIM framework.

An interpolation algorithm was implemented in the KIM platform to maintain marker tracking when one or more markers were temporarily lost with a confidence score < 0.6. For each frame, KIM first attempted to localize each marker directly within its tracking window. If a marker was not detected in a single frame, the tracking‐window center was retained at the marker's last known position. If the marker remained undetected for more than two consecutive frames, the expected marker position was estimated using the displacement of the remaining detected markers. If all markers could not be detected, then the marker positions were estimated from the marker history from two previous time points. If the marker detection looks incorrect, the user has the option to stop the treatment to manually segment the marker as a fallback strategy. For CNN‐based KIM, this scenario did not occur.

The extracted 2D information was subsequently input into KIM's 2D to 3D probability distribution function to generate 3D motion traces.^4^ However, evaluation of the generated 3D motion traces was beyond the scope of the present study.

#### 2.5.2 Search window initialization

Data obtained from the identification of marker positions during patients’ planning Cone Beam Computed Tomography (CBCT) was used to calculate a centroid for each marker based on its visible position within the three‐dimensional imaging plane. Following registration of centroid positions with the patient's anatomy, the expected region for each marker was defined around the planning centroid position to account for physiological motion, such as respiratory patterns. These centroid regions were used to initialize the marker detection window position.

### 2.6 Ground truth definition

Ground truth marker positions were manually generated using a custom MATLAB UI developed to enable labelling and recording of marker positions in sub‐cropped projections of a 120 pixel window located around the location predicted by the centroid projection. Though training images were automatically generated from existing logs to create a large dataset suitable for machine learning, the higher requirement for precision in testing necessitated manual detection of the marker positions. Although manual screening of all images in the large‐scale dataset was not feasible due to resource constraints, annotated multicenter, multiplatform datasets were created across 4184 images for 16 patients and 55 fractions from three centers to represent markers at every 10 degrees of gantry rotation. Images were manually segmented every 10 degrees of gantry rotation in a treatment arc without prior knowledge of the AI‐detected marker position, and all manual detections were visually verified to assess the ground truth. This visual assessment was performed independently of the labelling process, and images containing discrepancies greater than 20 pixels were relabeled.

The 46‐pixel search region was selected empirically during CNN‐KIM integration. This window size was chosen to encompass expected residual uncertainty around the projected marker centroid after CBCT‐based initialization and respiratory motion, while limiting the search area sufficiently to reduce false‐positive detections from nearby anatomical structures and maintain real‐time computation.

Within each 46‐pixel KIM search region, 32 × 32 pixel candidate tiles were extracted using a sliding‐window stride of 2 pixels. This generated overlapping candidate tiles centered at different candidate marker positions. When more than one candidate tile was classified as marker‐containing, the center coordinates of all positive tiles within the search region were averaged to determine a single marker centroid position. The resulting centroid was recorded as the detected marker position and passed to KIM for motion‐trace generation. When classification confidence was low (< 0.6 score), the detection was flagged as invalid and KIM used the interpolation strategy described below to maintain tracking continuity.

### 2.7 CNN evaluation

CNN performance was evaluated at two levels: tile‐level classification performance, quantified using sensitivity, specificity, and precision‐recall AUC for marker versus background candidate tiles, and integrated CNN‐KIM performance, quantified using marker‐centroid detection error. Marker‐centroid detection error was defined as the difference between the CNN‐KIM detected marker position and the manually defined ground‐truth marker position in the X and Y image dimensions, and was used as the primary clinical feasibility endpoint.

Latency was defined as the total time required for the CNN‐KIM workflow to process one kV image and generate marker detection output. This included image loading, tracking‐window generation, sliding‐window tile classification, centroid calculation, interpolation triggering when required, and output generation. Latency was measured using a timer implemented within the code, with the recorded value representing the elapsed time from image input to completion of marker detection for the whole image.

The robustness of the CNN model was further tested by examining detection error across three clinical centers using Elekta and Varian linacs, including data recorded from free‐breathing, deep‐inspiration breath‐hold, and end‐expiration breath‐hold treatments. To account for the absence of strong noise and scatter within the ground truth dataset, the KIM output log framework was modified to include a correlation parameter to indicate failure to detect a marker in the assigned marker detection window, necessitating interpolation.

### 2.8 Definition of feasibility for clinical implementation

Feasibility for clinical implementation was predefined using a study‐specific positional accuracy and processing‐time criterion, as justified below.

Although there are no specific AAPM Task Groups on the radiographic detection of markers, Task Groups TG‐142, TG‐147,^27^ TG‐135.B,^28^ and TG‐264^29^ were reviewed. From this review, feasibility for clinical applicability in this study was predefined as > 95% of marker positions being within 2 mm of the manually defined ground truth in each image dimension, together with a computation time below 150 ms per image. The > 2 mm criterion used in this study should be interpreted as a component‐level feasibility threshold for CNN‐KIM marker detection, rather than a complete end‐to‐end patient setup accuracy requirement.

AAPM Task Group 264 states that the average latency of the position monitoring signal for real time tracking should meet the definition of real‐time (≤ 500 ms).^29^ The overall KIM system latency was measured in 2014 to be 350 ms including the template‐based matching.^30^ Therefore, the computation time limit for the CNN marker detection is conservatively < 150 ms.

## 3 RESULTS

The CNN was evaluated for tile‐level classification of candidate image tiles and for marker‐centroid detection within the simulated real‐time KIM framework.

### 3.1 Evaluation of CNN classification

Analysis of false positives and false negatives revealed characteristic classification behavior, represented in Figure 3. False positives (FP) were constituted of marker‐like objects distinguished by the presence of high intensity foreground pixels with strong delineation against the background but excluded bony anatomy. Surgical clips were detected in the analysis of one patient. False negatives (FN) were primarily associated with poor background contrast, resultant from noise during the activation of the MV treatment beam, projection of markers onto radiopaque respiratory motion management blocks, and increased photon scattering at lateral angles. In contrast to the bimodal distribution of low dynamic range pixel intensities within correctly classified marker tiles, most FN classifications were characterized by a high dynamic range.

**FIGURE 3:**
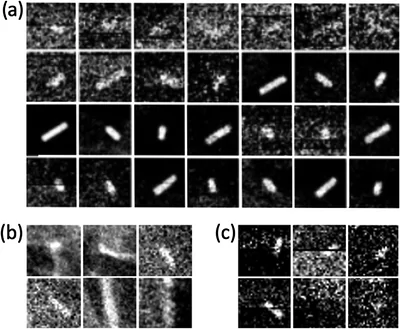
Examples of subtiles classified by CNN as (a) True positives, which indicates correctly detected markers, (b) False positives, which indicates background incorrectly classified as markers, and (c) False negatives, which indicates markers incorrectly classified as background.

The CNN demonstrated high accuracy in classifying markers from background images present within sub‐cropped kV image tiles, with the AUC of the PR curve being 0.9964. Sensitivity was 97.76%, while specificity was 99.94%. The CNN achieved a true positive rate of 97.75% and true negative rate of 99.94%, with a false positive rate of 0.059% and false negative rate of 2.25%. As background tiles substantially outnumbered marker‐containing tiles, tile‐level specificity of 99.94% should not be interpreted as the image‐level false‐positive rate. The image‐level false‐positive rate was 0.00252 false positives per image.

### 3.2 Marker detection accuracy and computation time

Visual validation indicated that the model was able to detect markers effectively across all breathing modes, producing two‐dimensional motion traces that closely adhered to the ground truth positions defined by manual detection within a sample fraction. Analysis of output logs from the implementation of the CNN demonstrated that detection accuracy was maintained within 2 mm thresholds in more than 95% of data points when compared to manually defined ground‐truth detections. Specifically, the model achieved sub‐2 mm accuracy in 98.3% of frames in the X‐direction and 99.2% in the Y‐direction. These results are visualized in Figure 4 and quantitatively summarized in Table 1. Gantry‐angle dependence of X‐ and Y‐direction detection error is shown in Figure 5. An example of the real‐time tracking framework is visualized in Figure 6.

**FIGURE 4:**
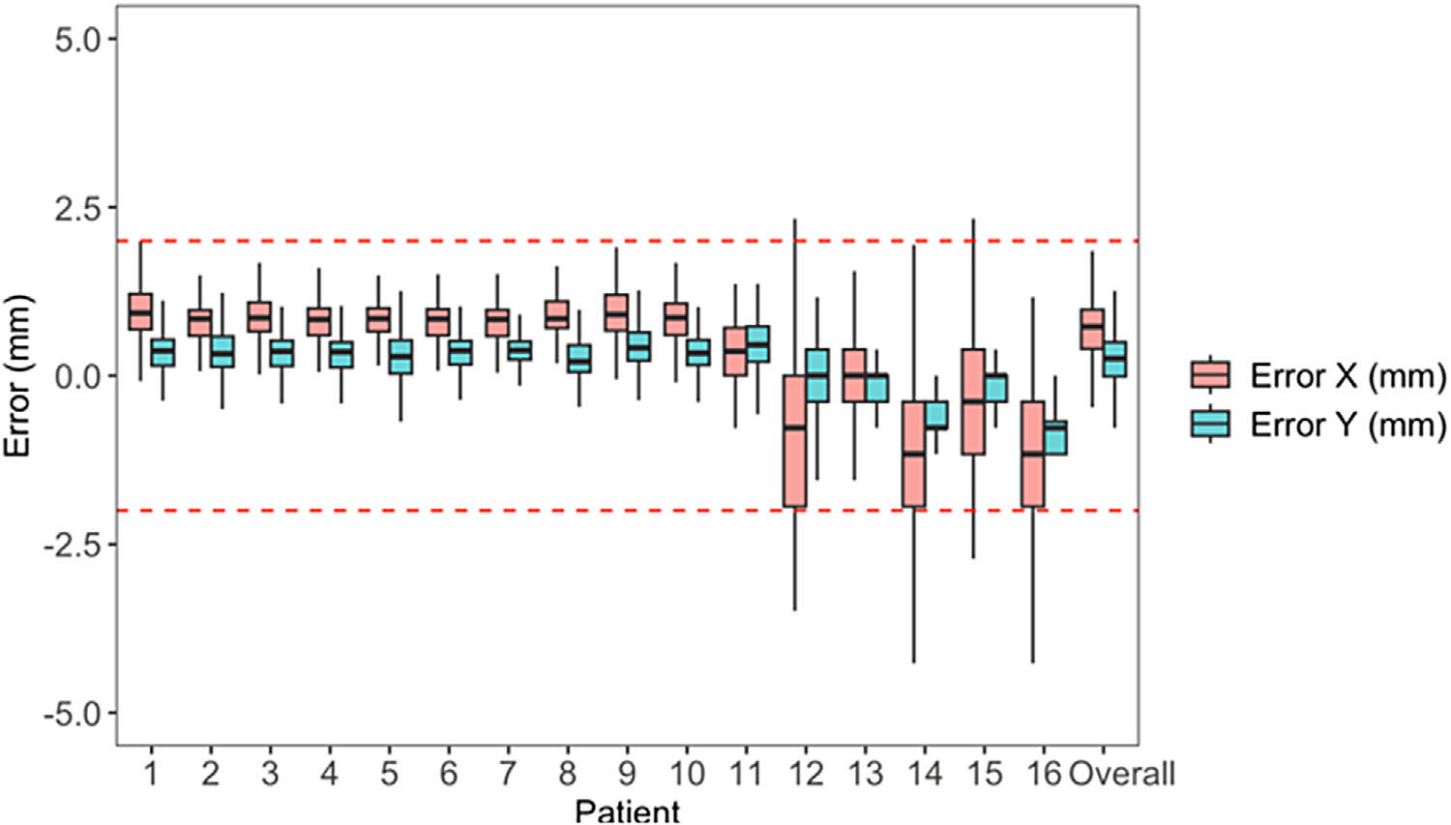
Boxplot of error in X and Y directions of real‐time CNN detections by patient when compared to ground truth definitions of marker position every 10 degrees of gantry rotation. The dashed red lines indicate the 2 mm threshold for error. Test set patients 1–11 belong to centre 1 (Varian), patients 12–14 belong to centre 2 (Elekta), patients 15–16 belong to Centre 3 (Elekta).

**TABLE 1:** Detection accuracy of CNN across lateral and anteroposterior angles error for CNN marker detection in X and Y directions within the real‐time KIM framework, compared against manually generated ground truth positions.

	Lateral (*n* = 1940)			Anteroposterior/posterior‐anterior (*n* = 2,244)		
	X	y	Euclidean	X	Y	Euclidean
Mean ± SD (mm)	0.8 ± 0.5	0.4 ± 0.6	1.1 ± 0.7	0.8 ± 0.5	0.4 ± 0.5	1.0 ± 0.6
Percentile (mm) [5th, 95th]	[0.2, 1.7]	[0.0, 1.1]	[0.4, 1.9]	[0.3, 1.7]	[0.0, 1.0]	[0.0, 1.9]
Max (mm)	3.8	2.8	3.8	3.6	3.1	3.8

**FIGURE 5:**
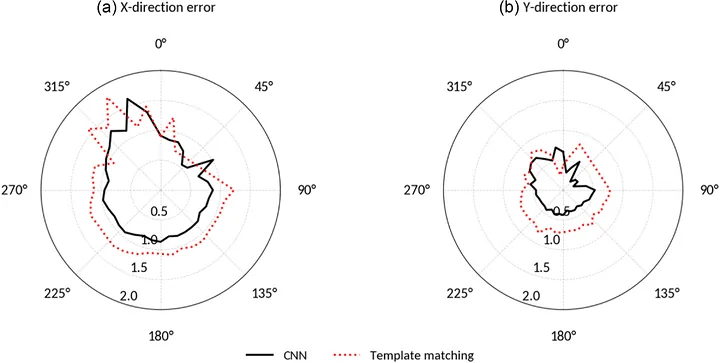
Gantry‐angle dependence of mean CNN‐based and template matching‐based localization errors (mm). Solid black lines represent CNN‐based localization and red dotted lines represent template matching.

**FIGURE 6:**
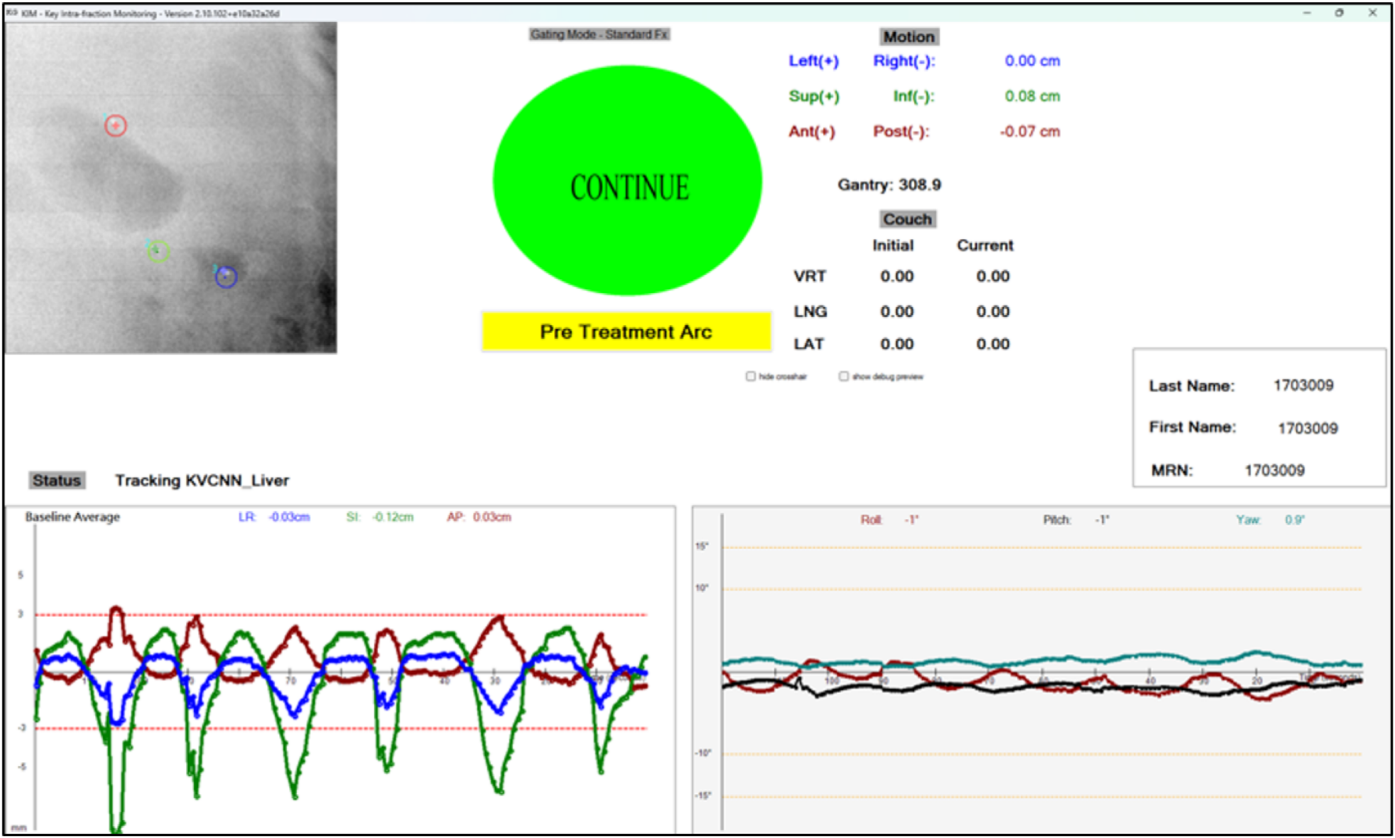
An example instance of motion tracking using the CNN model within the real‐time KIM framework on images collected from a liver SABR patient treated in free breathing. The top panel shows the X‐ray image and marker detections generated using the kV CNN developed in this work. The bottom panel shows the 3D tumor motion estimated using KIM's 2D‐to‐3D method,^4^ with motion displayed in the left‐right (blue), superior‐inferior (green), and anterior‐posterior (red) directions.

In paired comparisons of template matching and CNN detection performance, median absolute X/Y detection errors were 1.1/0.6 mm for template matching and 0.8/0.3 mm for CNN in the KIM framework. The 95th percentiles were 3.9/3.3 mm for template matching and 1.5/0.9 mm for the CNN. The proportion within 2 mm was 84.6% versus 98.3% in X and 92.2% versus 99.2% in Y for template matching and CNN, respectively. A demonstration video of the CNN operating within KIM is supplied in the supplementary material.

Within the simulated real‐time KIM system, processing time for kV images containing three markers ranged between 50–60 ms per image on an NVIDIA GeForce RTX 3070 GPU. For comparison, CPU execution on an Intel(R) Core(TM) i7‐7700HQ processor averaged 159.23 ms per frame for breath‐hold fractions and 144.4 ms per frame for free‐breathing fractions. The reported processing time of 50–60 ms per frame was measured by setting a timer within the code and recording the elapsed time from the program output.

Outlier behavior was most associated with MV beam activation, which increased noise and scatter in the kV images and caused transient marker detection disruptions within KIM. When marker occlusion occurred, the system occasionally transitioned to detecting a single marker based on its planned centroid projection, producing short sequences of error. In one region containing surgical clips near the planned centroid location, clips were misidentified as markers.

Dynamic interpolation was used to maintain continuous marker detection through these brief failures.

Performance varied significantly across different treatment centers (Table 2). Statistical analysis using the Kruskal‐Wallis rank sum test confirmed significant differences in error distributions across hospitals for both X and Y directions (df = 2, X: χ^2^ = 1539.5, *p* < 2.2 × 10^−^^1^ ^6^; Y: χ^2^ = 1515.9, *p* < 2.2 × 10^−^^1^ ^6^), and Post‐hoc Dunn's test further revealed significant pairwise difference between all centers, with adjusted p‐values less than 0.001. Notably, subtiles generated between centers were observed to differ in their pixel intensity distributions.

**TABLE 2:** Multi‐platform and multi‐breathing‐mode detection accuracy of CNN marker detection in X and Y directions within the real‐time KIM framework, compared against manually generated ground truth positions.

Dataset	X error (mm), mean ± SD	Y error (mm), mean ± SD	X 95th percentile (mm)	Y 95th percentile (mm)	Analysis context
Varian	0.8 ± 0.4	0.4 ± 0.5	1.5	0.9	Multi‐platform
Elekta	1.1 ± 1.1	0.7 ± 0.9	3.1	2.3	Multi‐platform
EEBH	0.9 ± 0.6	0.5 ± 0.6	1.9	1.2	Multi‐breathing mode
DIBH	0.8 ± 0.4	0.4 ± 0.3	1.6	0.9	Multi‐breathing mode
FB	0.8 ± 0.6	0.5 ± 0.9	1.4	1.4	Multi‐breathing mode

Images acquired from Centre 1 patients exhibited the lowest margins of error, and foreground images are characterized by a bimodal intensity distribution with low dynamic ranges. The clinically acceptable threshold (< 2 mm) for error was achieved in 98.3% of cases in the X direction, and 99.2% in the Y direction. Centre 2 represented the lowest detection accuracy in X and Y directions, with 83.4% of detections achieving a clinically acceptable threshold (< 2 mm) in the X direction, and 93.3% in the Y direction. For Centre 3 patients, the detection yielded clinical accuracy of 72.4% in the X direction and 92.5% in the Y direction. Subtiles derived from the X‐ray images of Centre 2 and Centre 3 patients represent a higher dynamic intensity range than Centre 1 subtiles, reflecting a lower contrast level between the image foreground (marker) and background. This higher proportion of pixel intensities in the upper quartiles reduced the visibility of markers against surrounding soft tissue structures.

## 4 DISCUSSION

In this study, we developed and evaluated a CNN‐based method for detecting fiducial markers in intrafraction kV images acquired during liver SABR. The proposed CNN method was then integrated into the KIM framework to evaluate its performance within the simulated real‐time KIM framework. Using kV images from the multicenter TROG 17.03 LARK clinical trial across Varian and Elekta linear accelerators, the method achieved the predefined study‐specific feasibility criterion for positional accuracy (Figure 4) in the simulated real‐time KIM system (Figure 6). These results show that CNN‐based marker detection can achieve clinically acceptable accuracy without requiring prior knowledge of marker appearance or additional imaging for template generation.

Previous applications of marker detection have either addressed organs with slow and small motion^8^, ^23^ or have been limited by the absence of patient datasets that leverage multicenter cooperation,^6^, ^22^ constraining the development of robust models capable of generalizing across imaging parameters present in different institutions. Here, the use of a large, annotated dataset spanning different motion‐management strategies and imaging systems allowed evaluation under clinically heterogeneous conditions. Maintaining sub‐2 mm accuracy (Table 1 and Figure 4) in more than 95% of assessed frames supports the potential robustness of the method across varied treatment settings. Although the CNN‐KIM detection results support feasibility, clinical deployment would require end‐to‐end quality assurance of the complete image‐guidance and motion‐management chain.

Our CNN model trained on corrected datasets demonstrated high sensitivity (97.76%) and specificity (99.94%), and reflected a comparable AUC with the uncorrected dataset, which included incorrect classifications of markers from the background because of template‐matching error within the KIM framework. The slight improvement in sensitivity between the uncorrected and corrected versions highlights that the model's capacity to reliably localize markers is increased by the refinement of training image classifications, despite a reduction in the overall size of the training set. However, our results indicated that while the presence of a low number of incorrect data labels did not substantially affect detection performance, it increased the time required for training convergence. This aligns with observations that as label corruption levels rise, convergence speed was diminished despite a minimal overall effect on model accuracy at lower corruption levels.^31^ As background tiles substantially outnumbered marker‐containing tiles, tile‐level specificity of 99.94% should not be interpreted as the image‐level false‐positive rate. The image‐level false‐positive rate was 0.00252 false positives per image.

Two‐way ANOVA testing of detection error values revealed that detection variability was influenced by both the clinical site and the individual patient. Treatment center (*p* < 0.001) and individual patient (*p* < 0.001) had a statistically significant effect on CNN detection error. These findings reinforce the impact of site‐specific imaging protocols and patient‐specific anatomical differences on model performance.

The observations were not uniformly sampled over the full gantry rotation. Of 4184 marker observations, 1940 (46.4%) were acquired at lateral kV projection angles and 2244 (53.6%) at AP/PA projection angles. This reflected both angular distribution in the treatment arc and image‐acquisition coverage, as well as a selection effect in the creation of the ground truth dataset. Manual ground‐truth annotation was only possible when fiducial markers remained sufficiently visible to be identified with confidence; images with severe MV scatter, noise, or complete marker obscuration were excluded to avoid introducing unreliable labels.

The CNN achieved a lower mean detection error on images from Varian machines (Patients 1–11) compared to Elekta machines (Patients 12–16), which were absent in training data (Figure 4). This difference was statistically significant (*t* = –8.94, *p* < 0.0001), and the reduced accuracy on Elekta‐acquired images reflects classification sensitivity to different levels of image contrast between vendors. Although the average error remained within the predefined threshold, the Elekta‐acquired images showed a wider error distribution and more outliers. One possible explanation is that the training dataset was biased toward Varian systems, resulting in better model generalization for Varian data. In addition, Elekta images had higher variability in acquisition settings across centers and generally lower contrast between markers and surrounding background structures, which may have increased classification uncertainty. There was no statistically significant difference between free‐breathing and breath‐hold treatments (Wilcoxon rank‐sum test, *p* = 0.13). This result should be interpreted cautiously, as only four free‐breathing patients were included, limiting the statistical power of the comparison.

We additionally acknowledge the unequal distribution of motion management techniques, with only four free‐breathing patients compared with 12 breath‐hold patients, and the lack of systematic augmentation for low‐contrast, high‐noise conditions. However, this distribution reflects real‐world clinical practice within the participating centers, where free‐breathing delivery is reserved for cases where breath‐hold is not feasible. Future work will focus on targeted data enrichment and augmentation strategies to better simulate low‐contrast, high‐noise scenarios and to further improve robustness across underrepresented motion management techniques. Although overfitting was reduced through patient‐level separation of training, validation, and testing data, data augmentation, early stopping, and use of a compact CNN architecture, the dataset remained imbalanced toward Centre 1 (Varian) imaging. The lower accuracy and increased outlier frequency observed in Elekta‐acquired images suggest that center‐ and vendor‐specific imaging characteristics influenced performance. Future development will require balanced multi‐vendor training data and may benefit from domain adaptation or vendor‐specific fine‐tuning.

Analysis of CNN performance compares favorably with previous applications of fully trained CNNs and models developed using transfer learning, which achieved sensitivities of 99.00% and 99.42% and specificity of 98.92% and 98.97% respectively.^8^ However, marker detection errors observed in our study (Table 1) reflect larger thresholds of error compared to the mean Euclidean and RMSE values reported in the application of a CNN to prostate cancer imaging.^8^ Specifically, previous studies^8^ achieved mean Euclidean errors and RMSE values along the X and Y axes that were lower than those observed in our liver marker study (Table 1). However, in contrast to the prostate, respiratory motion causes complex, multidimensional shifts of liver tumors that increase the potential for variability in marker position, leading to greater detection challenges.^4^ Nonetheless, the maintenance of clinically acceptable accuracy despite this variability demonstrates the robustness of the CNN model in dynamic imaging environments.

CNN performance in diverse datasets reflected a trade‐off within training and fine‐tuning to handle challenging imaging conditions.^6^ Though sensitivity could be increased by the introduction of high noise levels and variable contrast, additional augmentation also increased the rates of false positives by causing the model to incorrectly classify background artifacts as markers. Models may benefit from being selective, prioritizing specificity over sensitivity in clinical contexts to reduce the likelihood of false positives, which can lead to erroneous system interventions.^22^ Our findings align with this perspective, as interpolation can be employed to mitigate the impact of intermittent information loss from false negatives. Therefore, it is advantageous for the model to be conservative in its classifications of low‐quality images and privilege a higher specificity at the expense of a slight reduction in sensitivity.

Integrated within a simulated real‐time KIM framework, our CNN processed kV images with three markers in approximately 159 ms per frame (free‐breathing) and 144 ms (breath‐hold) on an Intel Core i7‐7700HQ CPU, reduced to 50–60 ms with a NVIDIA GeForce RTX 3070 GPU. This processing speed compares favorably with benchmarks set for detection speeds in prior studies^7^, ^8^, ^22^ aligning with the sub‐500 ms latency requirement for real‐time clinical tracking during treatment delivery^5^, ^8^, ^32^ following AAPM Task Group 264^29^ recommendations. This performance is compatible with standard linac frame rates of 5–10 fps, with potential for up to 15 fps on Varian systems and nominal rates of 5.5 fps on Elekta systems^33^ demonstrating the suitability of our CNN for efficient and accurate IGRT intrafraction monitoring.

In comparison to template matching in KIM, template matching often fails in regions with complex anatomical structures or in the presence of noise and artefacts such as surgical clips, necessitating interruption of treatment for template regeneration, which additionally increases effective radiation dose. In paired comparisons of template matching and CNN detection performance, median absolute X/Y detection errors were 1.1/0.6 mm for template matching and 0.8/0.3 mm for CNN in the KIM framework. The 95th percentiles were 3.9/3.3 mm for template matching and 1.5/0.9 mm for the CNN. The proportion within 2 mm was 84.6% versus 98.3% in X and 92.2% versus 99.2% in Y for template matching and CNN, respectively. Template matching performed similarly or better than the CNN at selected favorable gantry angles where the marker appearance was sharp, isolated, and similar to the reference template, with minimal overlap from anatomy, other markers, or treatment hardware. However, the CNN reduced the overall error distribution and improved robustness in more challenging frames with marker overlap, image noise, changing projection geometry, and partial obscuration.

The ground‐truth generation process may introduce selection bias due to the exclusion of images with uncertain marker visibility. However, this approach is consistent with established practice in medical imaging and machine learning studies,^34^, ^35^ where high‐confidence annotations are prioritized to ensure reference standard validity and to avoid propagation of label noise. While this improves the reliability of the reference dataset, it may introduce a bias toward higher‐quality images and should be considered when interpreting the reported detection accuracy. Manual detection was intentionally not performed for images in which the markers were not visually identifiable due to severe MV scatter or image noise. While this results in an underrepresentation of challenging lateral‐angle images, excluding such cases was considered preferable to introducing potentially inaccurate labels into the dataset. When marker visibility is insufficient for reliable human identification, any manually assigned annotation would be highly uncertain and could distort both training and evaluation. In principle, several approaches can be used to reduce noise in kV images. Hardware‐based strategies include synchronizing image acquisition with the pulsed beam, such as triggered imaging on Varian systems, and the use of anti‐scatter grids to reduce scattered radiation reaching the detector. Software‐based approaches include digital denoising filters and physics‐based methods, such as Monte Carlo simulations, to estimate and compensate for scatter. Future work could investigate these techniques to improve marker visibility in low‐contrast and high‐scatter projections.

This work focused on improving 2D detection of the markers; however, extension to 3D target trajectory reconstruction can be achieved through integration of multiple imaging projections and existing reconstruction frameworks.^4^ In particular, the KIM framework (Figure 6) enables reconstruction of 3D and 6D target positions using a Gaussian probability density function (PDF)‐based approach.^4^ Furthermore, rotational motion of the markers can also be extracted within the KIM framework, although this was not evaluated in the present study.

Future approaches can additionally leverage Region‐based Convolutional Neural Networks (RCNNs) to generate synthetic kV images to assist models in learning domain invariant features,^36^ which can help mitigate center‐specific variations in performance observed in this study. Although many existing frameworks trained on simulated datasets often fail to replicate the nuances of clinical images, integrating RCNNs demonstrates potential to balance datasets and enhance the robustness of models across different imaging conditions.^6^, ^36^, ^37^

Despite potential delays in treatment and associated risks of the percutaneous procedure of implantation, markers still potentially confer a benefit in guiding stereotactic liver radiation, especially for lesions close to radiosensitive organs such as small bowel.^38^ When considering marker‐based versus markerless tracking, markerless methods may exhibit reduced accuracy compared with marker‐based approaches, particularly in challenging imaging conditions or overlapping anatomical structures. This trend is consistent with the inherent reliance of markerless methods on indirect anatomical surrogates rather than high‐contrast fiducial signals. At the same time, markerless tracking offers an important clinical advantage by enabling a fully non‐invasive, surgical‐free workflow, thereby avoiding the risks associated with marker implantation. This represents a critical balance between invasiveness and tracking precision: marker‐based methods typically provide superior detection accuracy, whereas markerless approaches prioritize patient comfort and procedural simplicity at the potential cost of reduced robustness. Future work should therefore focus on stratifying patients based on suitability for markerless tracking, with the goal of identifying cases where markerless performance is insufficient for clinical requirements. In such scenarios, selective marker implantation may still be warranted to ensure tracking reliability. This hybrid strategy may enable an optimal balance between non‐invasive workflow design and the need for high‐precision motion tracking in radiation therapy applications.

## 5 CONCLUSION

In this study, we developed and validated a real‐time deep learning framework for detecting markers in kV images during liver SABR, fulfilling our aim by achieving clinically relevant CNN marker detection accuracy across a multi‐institutional, multiplatform dataset. Our model addresses existing limitations of template matching by eliminating the need for additional imaging or prior knowledge of marker properties. Sustained detection performance across different motion management strategies in both Varian and Elekta treatment systems indicates strong potential for the deep learning framework to improve radiation delivery accuracy in clinical settings. Despite variations in image quality, it consistently maintained sub‐2 mm precision in over 95% of frames, meeting the study‐specific feasibility criterion for clinical integration. Future work will additionally address cross‐center imaging variations and explore markerless tracking for a less invasive real‐time tumor monitoring approach.

## CONFLICT OF INTEREST STATEMENT

The authors declare the following financial interests/personal relationships which may be considered as potential competing interests: [P.J. Keall is an inventor on a patent related to the KIM technology that is licensed to Varian Medical Systems by Stanford University. Authors P.J. Keall and D.T. Nguyen are inventors on additional patents/patent applications related to the KIM technology that have been assigned to the company SeeTreat. P.J. Keall and D.T. Nguyen are founders and directors of SeeTreat. N. Hardcastle consults for SeeTreat.]

## CONSENT STATEMENT

Informed consent was obtained before recruiting patients in this clinical trial. The privacy rights of human subjects were always observed.
